# Clinicopathologic and prognostic significance of C-reactive protein/albumin ratio in patients with solid tumors: an updated systemic review and meta-analysis

**DOI:** 10.18632/oncotarget.24172

**Published:** 2018-01-11

**Authors:** Jiayuan Wu, Wenkai Tan, Lin Chen, Zhe Huang, Shao Mai

**Affiliations:** ^1^ Clinical Research Center, The Affiliated Hospital of Guangdong Medical University, Zhanjiang 524001, Guangdong Province, PR China; ^2^ Department of Gastroenterology, The Affiliated Hospital of Guangdong Medical University, Zhanjiang 524001, Guangdong Province, PR China; ^3^ Department of Cardiac Surgery, The Affiliated Hospital of Guangdong Medical University, Zhanjiang 524001, Guangdong Province, PR China; ^4^ Department of Gastrointestinal Surgery, The Affiliated Hospital of Guangdong Medical University, Zhanjiang 524001, Guangdong Province, PR China

**Keywords:** C-reactive protein/albumin ratio, prognosis, clinicopathological features, solid tumors, undated meta-analysis

## Abstract

C-reactive protein/albumin ratio (CAR) was originally used as a novel inflammation-based prognostic score in predicting outcomes in septic patients. Recently, more and more studies have reported the prognostic value of pretreatment CAR in solid tumors. However, the results remain controversial rather than conclusive. We conducted a meta-analysis based on 24 studies with 10203 patients to explore the relationship between CAR and survival outcomes in patients with solid tumors. The correlation between CAR and clinicopathological parameters was also assessed. Hazard ratio (HR) or odds ratio (OR) with its 95% confidence interval (CI) was applied to be the effect size estimate. The overall results showed that elevated CAR was associated with shorter overall survival (OS) (including 23 studies and 10067 patients) and poorer disease-free survival (DFS) (including 6 studies and 2904 patients). Significant associations between high CAR level and poor OS were also found in the subgroup analyses of study region, cancer type, primary treatment, clinical stage, cut-off selection, sample size, and cut-off value. Moreover, subgroup analyses demonstrated that study region, primary treatment, clinical stage, sample size, and cut-off value did not alter the prognostic value of CAR for DFS. Furthermore, elevated CAR was correlated with certain phenotypes of tumor aggressiveness, such as poor histological grade, serious clinical stage, advanced tumor depth, positive lymph node metastasis, and positive distant metastasis. Together, our meta-analysis suggests that elevated level of serum CAR predicts worse survival and unfavorable clinical characteristics in cancer patients, and CAR may serve as an effective prognostic factor for solid tumors.

## INTRODUCTION

In 1863, Rudolf Virchow first provided the hypothesis of a possible correlation between inflammation and malignant tumor according to the presence of leukocytes within tumors [1]. Yet, it is only during the last twenty years that clear evidence has been obtained that inflammation plays a decisive role in carcinogenesis and tumor progression; for example, inflammation regulates tumor behavior at different stages of tumor development, including initiation, promotion, malignant conversion, invasion, and metastasis [2]. Probable mechanisms might be that inflammation could result in malnutrition, immune dysfunction, up-regulation of growth factors, and angiogenesis. Lately, epidemiological studies have revealed that infection-related inflammation contributes to approximately 20% of all cancer cases worldwide, and chronic inflammation increases the risk of human cancers of almost all organs/tissues, which predisposes individuals to various types of cancers [3]. Several inflammation-inducible factors, such as air pollution, foreign bodies and ultraviolet radiation, are also associated with carcinogenesis [4]. In some cases, inflammatory cells can help to form a tumor microenvironment, which is an indispensable actor in the neoplastic process. Moreover, increasing evidences have proved that anti-inflammatory therapy can reduce the risk and prolong the survival of patients in some cancer types, such as ovarian cancer, cervical cancer, and gastrointestinal malignances, which further supports the close connection between inflammation and cancer development [5, 6]. Thus, the understanding of cancer-related inflammation could provide novel and alternative strategies for biological intervention of malignant tumors. On the other hand, malnutrition is common in patients with solid tumors, especially in progressive stage. Malnutrition can weaken a number of defense mechanisms in human body, including physiologic barrier, immune system, and phagocyte function. This problem has been proved to be correlated with increased susceptibility to infection, poor curative response, serious therapeutic side effects, and worse survival [7]. Several studies have showed that nutritional interventions can help cancer patients to maintain body weight, improved quality of life, and decrease mortality [8, 9]. Therefore, it is critical to precisely and early identify nutritional risk in cancer patients.

It is a consensus that a pretreatment and effective parameter to evaluate survival probability and prognosis is necessary for decision-making concerning clinical therapy of malignant tumors. Traditionally, the tumor-nodes-metastasis (TNM) staging system has been the most common tool to predict clinical outcomes and to formulate rational treatment strategies for patients with solid tumors [10]. Nevertheless, patients with the same clinical stage often present large variations in clinical outcomes, especially in some cases with advanced disease. Moreover, TNM staging does not fully consider many essential variables, including the biological variability of the tumor itself, patients’ characteristics, laboratory test, and treatment approach, thus, it is inaccurate for predicting prognosis [11]. Except the tumor stage, some other histopathological indicators have been reported to be oncological prognostic indicators by previous studies, such as tumor size, histologic grade, and vascular or nodal involvement, but these variables can only be evaluated after surgical exploration [12]. Therefore, it is urgent to look for reliable prognostic markers for better risk stratification and optimal therapeutic plans of cancer patients.

Given the close relationship between inflammation and cancer, several inflammation- based prognostic systems have been developed to predict the clinical outcome during the course from bench to bedside. These factors, including C-reactive protein (CRP), Glasgow Prognostic Score (GPS), modified Glasgow Prognostic Score (mGPS), high-sensitivity modified Glasgow prognostic score (HS-mGPS), neutrophil to lymphocyte ratio (NLR), platelet to lymphocyte ratio (PLR), and systemic immune-inflammation index (SII), are all easily obtainable from peripheral blood samples, and have been validated in many types of cancer [13–15]. Additionally, not only the physical and metabolic effects of the disease but also the effects of anticancer treatment can lead to inadequate food intake, decreased physical activity, and catabolic metabolic derangement, resulting in cancer patients at risk of malnutrition [16]. Serum albumin (ALB) level is closely related to the degree of malnutrition, so it is commonly used as an indicator of nutritional status. Furthermore, numerous studies have suggested that the lower the level of serum ALB, the worse the prognosis of cancer patients [17, 18]. Thus, assessment of serum ALB has emerged as a potential prognostic factor in various cancers, since nutritional status can be corrected prior to therapy.

The C-reactive protein/albumin ratio (CAR), represented as a combination of serum CRP and ALB counts, was initially used as a new inflammation-based prognostic score for the purpose of predicting mortality in patients with sepsis [19]. CAR indicates the balance of the inflammatory and nutritional status, also making it a useful index for predicting prognosis in malignance. Recently, numerous studies reported that elevated CAR level was associated with poor prognosis in solid tumors. However, some studies revealed conflicting findings due to the variance in study design, sample size and patient feature. Although a meta-analysis on this topic has been published, only 10 publications were included in that meta-analysis [20]. There have been more than 14 papers continuously published since this meta-analysis was conducted. Thus, the real value of CAR in predicting prognosis of solid tumors has not yet been fully elucidated. For these reasons, we embarked on this update meta-analysis to derive a more precise estimation of the prognostic impact of CAR in patients with solid tumors.

## RESULTS

### Description of included studies

**Figure 1:**
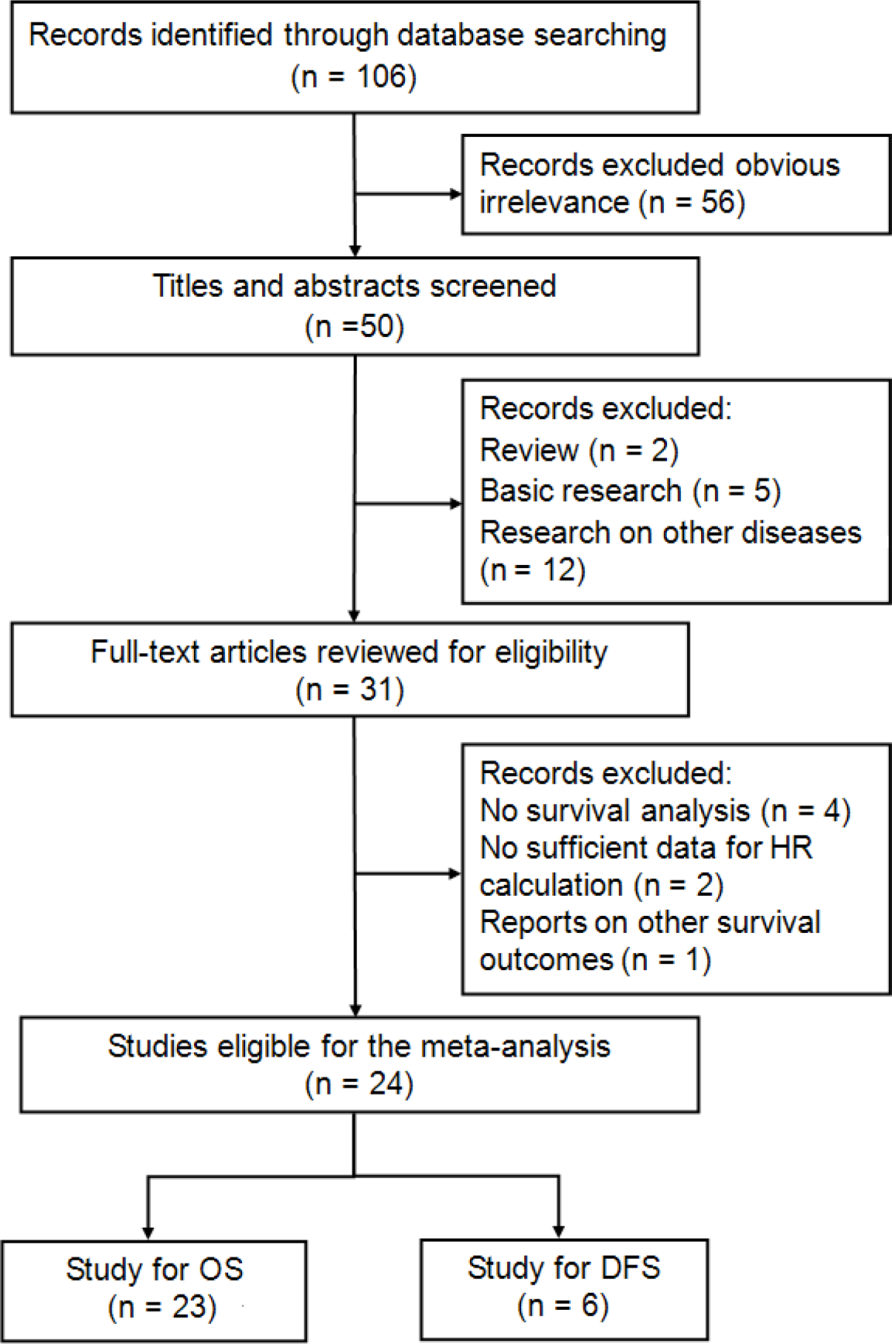
Flow diagram of the study selection process and specific reasons for exclusion in the meta-analysis

The process of literature search was shown in Figure 1. Initially, 106 papers were generated in the primary electronic search in the major databases. Twenty-four full-text articles published from 2015 to 2017 met the inclusion criteria and were selected for our meta-analysis [21–44]. A total of 10203 patients diagnosed with various cancers, including laryngeal squamous cell carcinoma (LSCC) [22], ovarian cancer (OC) [23], renal cell carcinoma (RCC) [24], non-small cell lung cancer (NSCLC) [25], pancreatic cancer (PC) [26–29], colorectal cancer (CRC) [30–33], nasopharyngeal cancer (NPC) [21, 34–36], gastric cancer (GC) [37, 42], oral squamous cell carcinoma (OSCC) [38], hepatocellular carcinoma (HCC) [39, 43], esophageal squamous cell carcinoma (ESCC) [40, 41], small cell lung cancer (SCLC) [44], were included. These studies originated from China [21–24, 27, 28, 33–36, 39–42, 44], Japan [26, 30–32, 37, 43], and Korea [25, 29, 38], respectively. Twenty-three articles reported the outcomes of overall survival (OS) [21–31, 33–44], and 6 studies presented disease free survival (DFS) as survival endpoint [22, 24, 26, 32, 34, 37]. Seventeen studies included cancer patients with all disease stages (Stage I–IV or limited-extensive stage) [22–26, 28–31, 34–36, 38, 39, 41, 43, 44], three studies recruited patients with Stage I–III [37, 40, 42], and four studies reported advanced stages (III, IV, or III-IV) [21, 27, 32, 33]. The primary treatments were extremely various among these 24 included studies, including surgery [22–24, 26, 31, 37–43], chemotherapy (CT) [21, 25, 27, 29, 30, 32, 33, 44], and concurrent chemoradiotherapy (CCRT) [34–36]. The hazard ratios (HRs) and the corresponding 95% confidence intervals (CIs) of these 24 studies were all directly extracted from the outcomes of multivariate analysis. The cut-off values of defining elevated CAR were calculated by the receiver operating characteristic (ROC) curve in 19 studies [22–24, 26, 28–35, 37–43], by cutoff finder in 4 studies [21, 25, 27, 44], and by median value in 1 study [36], which ranged from 0.03 to 0.68, and were extremely different among the including studies. According to the quality criteria, all cohort studies had scores of six or more and were of high quality. The main characteristics of the included studies were listed in Supplementary Table 1.

### Association of CAR with OS

**Figure 2:**
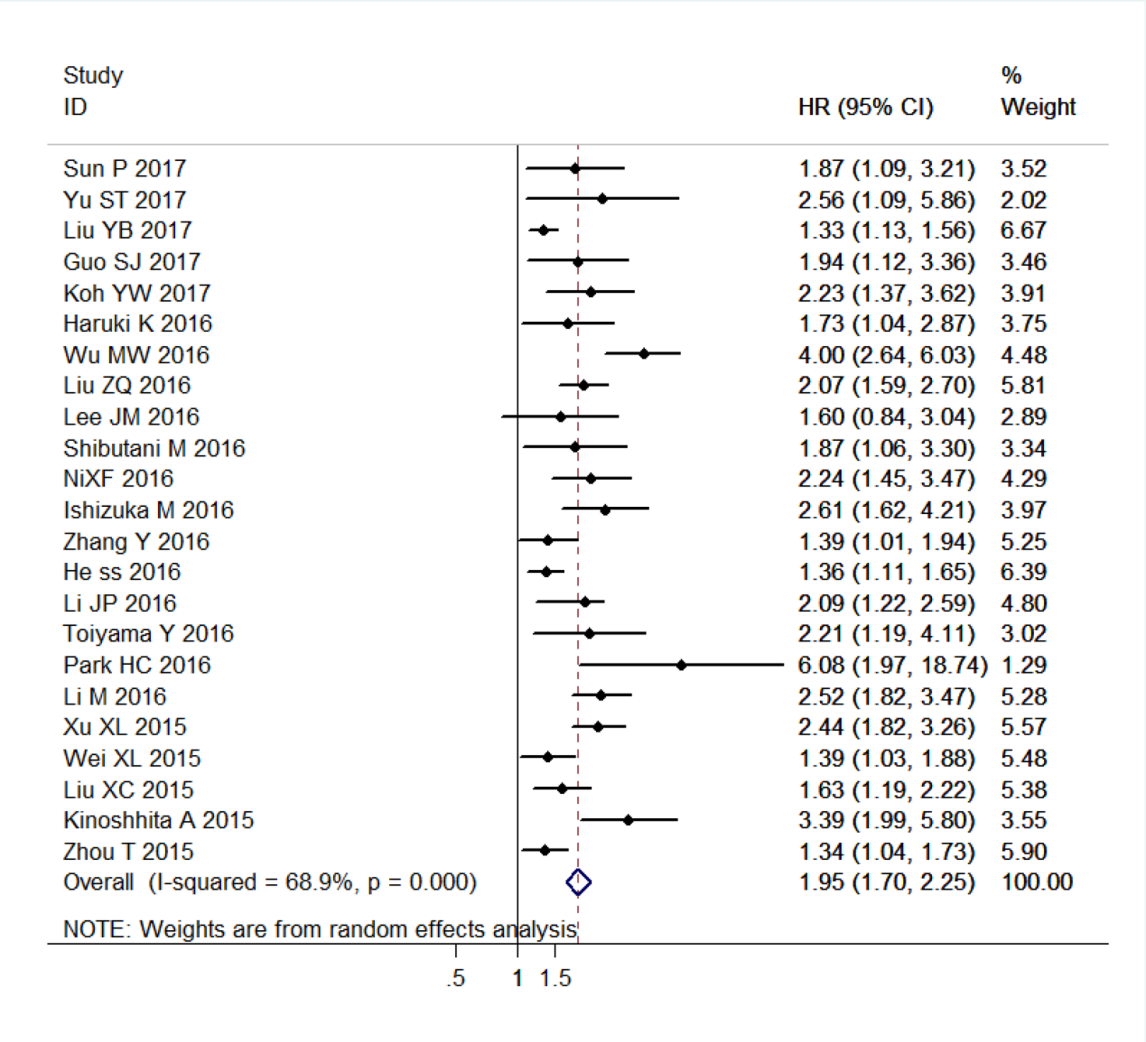
Forest plots of the association between C-reactive protein/albumin ratio and overall survival in patients with solid tumors Abbreviation: HR hazard ratio; CI confidence interval.

The combined analysis of 23 studies with 10067 patients showed that patients with elevated CAR were expected to suffer unfavorable OS (HR = 1.95, 95% CI: 1.70–2.25, *P* < 0.001, random effects; Figure 2). When stratified by cancer type, high CAR predicted poor OS for patients with PC (HR = 2.25, 95% CI: 1.52–3.34, *P* < 0.001, random effects), CRC (HR = 2.26, 95% CI: 1.71–2.99, *P* < 0.001, fixed effects), NPC (HR = 1.50, 95% CI: 1.29–1.74, *P* < 0.001, random effects), ESCC (HR = 1.84, 95% CI: 1.06–3.20, *P* = 0.030, random effects), GC (HR = 1.73, 95% CI: 1.31–2.29, *P* < 0.001, fixed effects), HCC (HR = 2.73, 95% CI: 2.07–3.60, *P* < 0.001, fixed effects), head and neck cancer (including LSCC and OSCC) (HR = 3.49, 95% CI: 1.78–6.85, *P* < 0.001, fixed effects), and other cancers (HR = 1.41, 95% CI: 1.24–1.60, *P* < 0.001, fixed effects). Similarly, when grouped based on study region, the prognostic role of elevated CAR in predicting shorter OS was obvious in studies originated from China (HR = 1.84, 95% CI: 1.57–2.17, *P* < 0.001, random effects), Japan (HR = 1.82, 95% CI: 1.60–1.85, *P* < 0.001, fixed effects), and Korea (HR = 2.39, 95% CI: 1.34–4.24, *P* = 0.003, random effects). Moreover, the significant association of high CAR and worse OS did not change regardless of the subgroup analyses of primary treatment, clinical stage, cut-off selection, sample size, and cut-off value (Table 1).

**Table 1:** Pooled and subgroup analyses of the main results for the meta-analysis of overall survival

Categories	*n*	Model	HR (95% CI)	*Z*	*P*	Heterogeneity	
						*I*^2^	*P*_h_
**Overall survival (OS)**	**23 (10067)**	**Random**	**1.95 (1.70–2.25)**	**9.33**	**< 0.001**	**68.9%**	**< 0.001**
Study region							
China	15 (8371)	Random	1.84 (1.57–2.17)	7.41	< 0.001	74.2%	< 0.001
Japan	5 (1409)	Fixed	1.72 (1.60–1.85)	6.85	< 0.001	0.6%	0.403
Korea	3 (287)	Random	2.39 (1.34–4.24)	2.96	0.003	50.9%	0.131
Cancer type							
PC	4 (814)	Random	2.25 (1.52–3.34)	4.04	< 0.001	69.8%	0.019
CRC	3 (874)	Fixed	2.26 (1.71–2.99)	5.70	< 0.001	0.0%	0.678
NPC	4 (4814)	Fixed	1.50 (1.29–1.74)	5.32	< 0.001	37.1%	0.189
ESCC	2 (891)	Random	1.84 (1.06–3.20)	2.18	0.030	85.6%	0.008
GC	2 (839)	Fixed	1.73 (1.31–2.29)	3.87	< 0.001	0.0%	0.390
HCC	2 (364)	Fixed	2.73 (2.07–3.60)	7.12	< 0.001	0.0%	0.352
HNC	2 (169)	Fixed	3.49 (1.78–6.85)	3.63	< 0.001	31.3%	0.228
Others	4 (1302)	Fixed	1.41 (1.24–1.60)	5.27	< 0.001	44.2%	0.146
Primary treatment							
Surgery	12 (3773)	Random	2.06 (1.66–2.55)	6.60	< 0.001	71.2%	< 0.001
CT	7 (1242)	Random	2.05 (1.49–2.81)	4.42	< 0.001	71.4%	0.002
CCRT	3 (4666)	Random	1.53 (1.20–1.94)	3.43	0.001	50.8%	0.131
NR	1 (386)	Random	2.07 (1.59–2.70)	5.39	< 0.001	NA	NA
Clinical stage							
All	17 (8231)	Random	1.82 (1.57–2.12)	7.82	< 0.001	64.4%	< 0.001
I – III	3 (1307)	Fixed	2.04 (1.67–2.50)	6.94	< 0.001	42.9%	0.173
Advanced	3 (529)	Random	2.61 (1.65–4.13)	4.10	< 0.001	66.4%	0.051
Cut-off selection							
ROC	18 (8745)	Random	1.90 (1.63–2.21)	8.37	< 0.001	65.1%	< 0.001
Cut-off finder	4 (913)	Random	2.15 (1.27–3.65)	2.86	0.004	85.1%	< 0.001
Median	1 (409)	Random	2.09 (1.43–3.05)	3.84	< 0.001	NA	NA
Sample size							
< 200	10 (1288)	Fixed	2.27 (1.93–2.68)	9.87	< 0.001	0.0%	0.459
≥ 200	13 (8779)	Random	1.81 (1.52–2.15)	6.76	< 0.001	75.5%	< 0.001
Cut-off value							
< 0.1	11 (7138)	Random	1.92 (1.55–2.37)	6.04	< 0.001	60.6%	0.005
≥ 0.1	12 (2929)	Random	1.98 (1.62–2.41)	6.77	< 0.001	75.4%	< 0.001

PC pancreatic cancer; CRC colorectal cancer; NPC nasopharyngeal carcinoma; ESCC esophageal squamous cell carcinoma; GC gastric cancer; HCC hepatocellular carcinoma; HNC head and neck cancer (including laryngeal squamous cell carcinoma and oral squamous cell carcinoma); CT chemotherapy; CCRT concurrent chemoradiotherapy; ROC the receiver operating characteristic.

*P* denotes *P* value for statistical significance based on *Z* test; *P*_h_ denotes *P* value for heterogeneity based on Q test. HR hazard ratio; CI confidence interval; NA not available.

The result of heterogeneity analysis illustrated that all of the included datasets of OS had extreme heterogeneity (I^2^ = 68.9%, *P*_h_ < 0.001). Thus, we used a random-effects model to estimate the overall HR for OS. When the subgroup analysis was conducted to assess the source of heterogeneity based on study region, cancer type, primary treatment, clinical stage, sample size, and cutoff value, the heterogeneity was slightly reduced in most subgroups, but remained statistically significant in several subgroups (Table 1).

### Association of CAR with DFS

**Figure 3:**
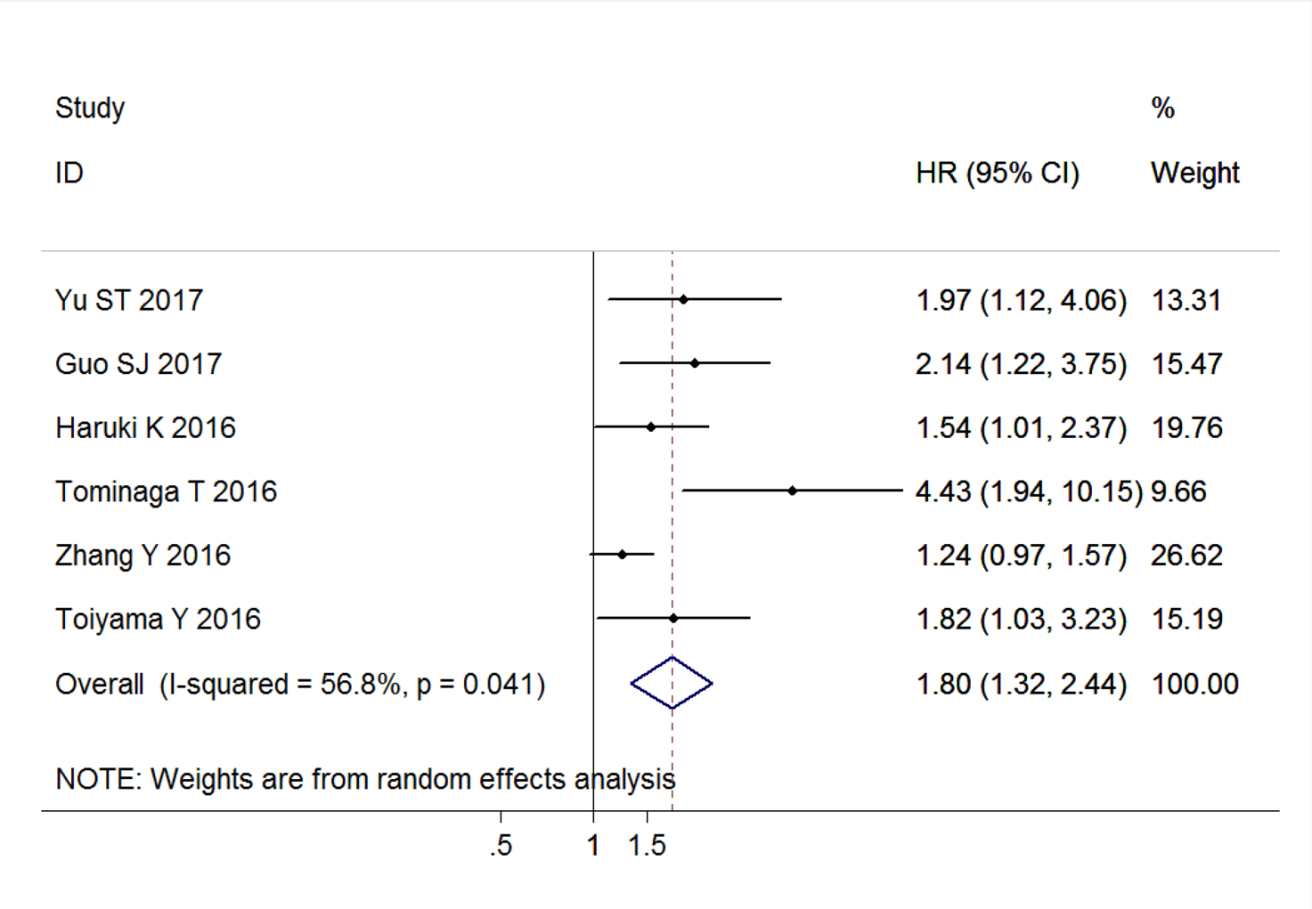
Forest plots of the association between C-reactive protein/albumin ratio and disease-free survival in patients with solid tumors Abbreviation: HR hazard ratio; CI confidence interval.

Six studies comprising 2904 patients reported the outcomes for DFS, and the pooled result indicated that elevated CAR was associated with poor DFS (HR = 1.80, 95% CI: 1.32–2.44, *P* < 0.001, random effects; Figure 3). This significant associations were also found in the subgroup analyses of China (HR = 1.60, 95% CI: 1.08–2.36, *P* = 0.018, random effects), and Japan (HR = 2.10, 95% CI: 1.23–3.59, *P* = 0.007, random effects). Similarly, the positive results were observed in the subgroup analyses based on primary treatment, clinical stage, sample size, and cut-off value (Table 2).

**Table 2:** Pooled and subgroup analyses of the main results for the meta-analysis of disease-free survival

Categories	*n*	Model	HR (95% CI)	*Z*	*P*	Heterogeneity	
						*I*^2^	*P*_h_
**Disease-free survival (DFS)**	**6 (2904)**	**Random**	**1.80 (1.32–2.44)**	**3.72**	**< 0.001**	**56.8%**	**0.041**
Study region							
China	3 (2271)	Random	1.60 (1.08–2.36)	2.36	0.018	52.9%	0.119
Japan	3 (633)	Random	2.10 (1.23–3.59)	2.72	0.007	59.8%	0.083
Primary treatment							
Surgery	4 (1196)	Fixed	1.79 (1.37–2.34)	4.30	< 0.001	0.0%	0.812
Others	2 (1708)	Random	1.37 (1.09–1.73)	2.67	0.008	88.1%	0.004
Clinical stage							
All	4 (2384)	Fixed	1.43 (1.19–1.73)	3.73	< 0.001	31.7%	0.222
Others	2 (520)	Random	2.70 (1.13–6.40)	2.25	0.025	66.7%	0.083
Sample size							
< 200	3 (378)	Random	2.18 (1.24–3.81)	2.72	0.006	59.6%	0.084
≥ 200	3 (2526)	Fixed	1.40 (1.14–1.73)	3.22	0.001	49.7%	0.137
Cut-off value							
< 0.1	5 (2768)	Fixed	1.46 (1.23–1.75)	4.19	< 0.001	20.2%	0.286
≥ 0.1	1 (136)	Random	4.30 (1.94–10.13)	3.53	< 0.001	NA	NA

*P* denotes *P* value for statistical significance based on *Z* test; *P*_h_ denotes *P* value for heterogeneity based on *Q* test. HR hazard ratio; CI confidence interval; NA not available.

An extreme heterogeneity was also found among the included studies of DFS (I^2^ = 56.8%, *P*_h_ = 0.041); thus, a random-effects model was applied to calculated the pooled outcome. When the subgroup analysis was conducted, the heterogeneity was obvious to be still significantly evident in most subgroups (Table 2).

### Association of CAR with clinicopathological characteristics

The relationship of CAR with clinicopathological features are illustrated in Table 3. Elevated CAR was correlated with certain phenotypes of tumor aggressiveness, such as poor histological grade (pooled odds ratio [OR] = 1.47; 95% CI = 1.14–1.90; *P* = 0.003; fixed effects), serious clinical stage (pooled OR = 3.20; 95% CI = 2.37–4.32; P < 0.001; random effects), advanced tumor depth (pooled OR = 2.57; 95% CI = 1.52–4.34; P < 0.001; random effects), positive lymph node metastasis (pooled OR = 2.225; 95% CI = 1.62–3.14; P < 0.001; random effects), and positive distant metastasis (pooled OR = 3.97; 95% CI = 1.56–10.09; P < 0.001; random effects). However, no association existed between CAR and smoking status (pooled OR = 1.16; 95% CI = 0.99–1.34; P = 0.052; fixed effects).

**Table 3:** Summary of the association of C-reactive protein/albumin ratio and clinopathological parameters in solid tumors

Category	*n*	Model	OR (95% CI)	*Z*	*P*	Heterogeneity	
						*I*^*2*^	*P*_*h*_
Histologic grade (poor vs. well or moderate)	7 (1983)	Fixed	1.47 (1.14–1.90)	2.97	0.003	0.0%	0.654
Clinical stage (III or IV vs. I or II)	12 (6279)	Random	3.20 (2.37–4.32)	7.58	< 0.001	71.8%	< 0.001
Tumor depth (T3+T4 vs. T1+T2)	7 (4367)	Random	2.57 (1.52–4.34)	3.52	< 0.001	80.0%	< 0.001
Lymph node metastasis (positive vs. negative)	8 (4496)	Random	2.25 (1.62–3.14)	4.83	< 0.001	60.5%	0.013
Distant metastasis (positive vs. negative)	4 (1266)	Random	3.97 (1.56–10.09)	2.89	0.004	75.8%	0.006
Smoking status (ever vs. never)	5 (3761)	Fixed	1.16 (1.00–1.34)	1.95	0.052	45.9%	0.117

*P* denotes *P* value for statistical significance based on *Z* test; *P*_h_ denotes *P* value for heterogeneity based on *Q* test. OR odds ratio; CI confidence interval.

### Sensitivity analysis and meta-regression analysis

**Figure 4:**
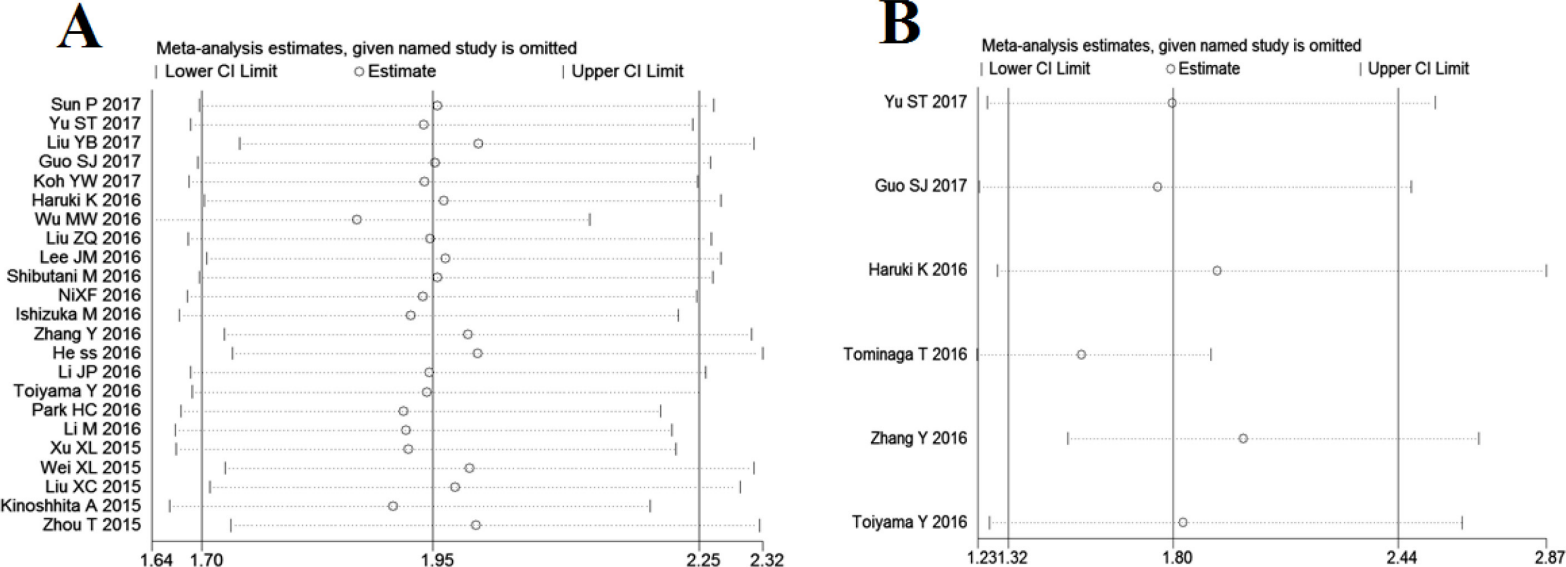
Effect of individual studies on pooled hazard ratios (HR) for the relationship between C-reactive protein/albumin ratio and prognosis of solid tumors (**A**) Sensitivity analysis for overall survival. (**B**) Sensitivity analysis for disease-free survival.

Sensitivity analysis was conducted to evaluate the robustness of association between CAR and survival outcomes, and the results suggested that no individual study significantly changed the overall HRs of our meta-analysis for OS and DFS (Figure 4).

We conducted meta-regression analysis to investigate the potential source of heterogeneity among studies for OS. In multivariate analysis, the results showed that study region (*P* = 0.387), cancer type (*P* = 0.710), primary treatment (*P* = 0.610), clinical stage (*P* = 0.234), sample size (*P* = 0.533), cut-off selection (0.666), and cut-off value (*P* = 0.498) did not contribute to the source of heterogeneity for OS. Due to the small number of included studies, we did not perform meta-regression analysis to explore the underlying source of heterogeneity among studies concerning DFS.

### Publication bias

**Figure 5:**
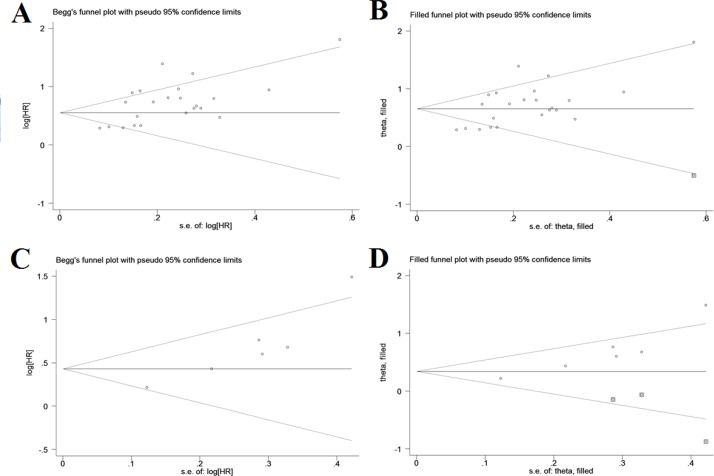
Funnel plots for assessment of potential publication bias in studies of C-reactive protein/albumin ratio and prognosis of solid tumors (**A**) Funnel plot of publication bias for studies reporting overall survival. (**B**) Funnel plot adjusted with trim and fill method for studies reporting overall survival. (**C**) Funnel plot of publication bias for studies reporting disease-free survival. (**D**) Funnel plot adjusted with trim and fill method for studies reporting disease-free survival.

Both Begg’s and Egger’s tests suggested a significant publication bias with regard to the pooled outcome of OS (Begg’s test: *P* = 0.098; Egger’s test: *P* = 0.001), and the funnel plot showed a certain degree of apparent asymmetry (Figure 5A), indicating potential publication bias. The trim-and-fill analysis showed that one more studies were needed to balance the funnel plot (Figure 5B). The adjusted HR and 95% CI were attenuated but remains significant (pooled HR = 1.93; 95% CI = 1.67–2.22; *P* < 0.001; random effects). Similarly, an obvious publication bias was also found concerning the pooled result of DFS (Begg’s test: *P* = 0.060; Egger’s test: *P* = 0.005; Figure 5C), and the HR estimate adjusted by the trim-and-fill analysis was still statistically significant after adding three unpublished studies (pooled HR = 1.41; 95% CI = 1.03–1.93; *P* = 0.035; random effects; Figure 5D), thereby suggesting that the potential publication bias had minimal impact on the overall outcomes.

## DISCUSSION

Although a first qualitative analysis of CAR levels related to prognostic outcome of various solid tumors has been published by Li et al. [20], there are still some shortcomings that may negatively impact the reliability of the final results. Firstly, only 10 studies and 4592 patients were included in the previous meta-analysis, the numbers of which were extremely small and the evidence-based power was not strong enough. Secondly, both the HRs of univariate and multivariate analyses were provided in the report of Wu et al. [27], but only the data of univariate analysis was retrieved in the previous meta-analysis, which might introduce some imprecision because it has been not adjusted by the confounding factors. Thirdly, previous study only reported the pooled result of OS, but not the overall outcome of DFS, which was also an important survival endpoint of malignant tumors. Finally, the data synthesis by Li et al. [20] failed to explore the relationship between CAR concentration and clinical characteristics, which might lead to lose some valuable information. Therefore, it is necessary to perform an updated meta-analysis to further investigate the relationship between CAR and prognosis in patients with solid tumors.

Our meta-analysis comprised 24 studies with 10203 patients, and the synthesized data suggested that elevated pretreatment CAR predicted unfavorable prognosis, including OS and DFS, in patients with solid tumors. In the subgroup analyses, the significant association between elevated CAR and poor survival did not observably changed. Thus, CAR could be an independent predictor of all-caused and disease-specific mortality in solid cancers. However, these overall results were along with extreme heterogeneity which might affect the analysis, interpretation, and conclusions of this study. Different baseline characteristic of individual studies, such as study region, cancer type, primary treatment, clinical stage, cut-off selection, sample size, and cut-off value, might lead to inter-study heterogeneity. Meta-regression analysis was performed by using the abovementioned factors to further explore the source of heterogeneity, but none of them could complain the heterogeneity. Instead, we used a random-effect model to minimize the effect of the heterogeneity on OS and DFS. Moreover, despite the broad search criteria, evidences of publication bias were found among the studies concerning OS and DFS, which might have inflated the pooled results. After recalculated by trim-and-fill analyses, the adjusted pooled effect size remained statistically. This finding indicated that the publication bias may not have a systematic influence on the pooled outcomes, and further confirmed the reliability of our results.

To further explore the prognostic impact of CAR on solid cancers, we analyzed the correlation between CAR and clinicopathological features. According to the pooled outcomes, high levels of CAR was significantly associated with several features of tumor progression, including poor histological grade, serious clinical stage, advanced tumor depth, positive lymph node metastasis, and positive distant metastasis. These results strongly support the predictive value of elevated CAR on worse prognosis in malignances, and high CAR is closely related to more aggressive tumor behavior. Therefore, cancer patients with above clinical features would be probably to benefit more from CAR estimation for clinical-decisions making.

Similar to CAR, the GPS/mGPS system are based on the serum levels of two acute phase proteins, CRP and ALB, but there are several fundamental differences between them. First, the GPS/mGPS system is an evaluation based on a three-point score (0, 1, or 2), making it have a qualitative nature with discontinuous values, while CAR is a simple ratio, deemed as a quantitative variable with a continuous value. Thus, CAR could provide more information than the GPS/mGPS system. Second, the GPS/mGPS system may overestimate or underestimate the inflammatory or nutritional level in body because it separately scores the serum levels of CRP and ALB, whereas CAR can decrease the potential risk of overestimated or underestimated results for the reason that it synthesize of the CRP and ALB values more reasonable [28, 43]. Third, as a continuous score, CAR has the ability to further distinguish the patients with the same score according to the GPS/mGPS system [27]. On the other hand, Liu et al. [23] reported that CAR, GPS, and mGPS were all predictors of survival in ovarian cancer by univariate analysis, but only CAR remained as an independent index for prognosis after adjusted by multivariate analysis, the result of which was the same to some reports on PC [26], NPC [36], and HCC [43]. Meanwhile, AUC analysis further identified the better predictive accuracy of CAR rather than other inflammation-based prognostic scores, including GPS and mGPS, which is consistent with several previous studies in CRC [33], ESCC [40, 41], GC [42] and HCC [43]. Therefore, the GPS/mGPS system exposed some defects in its clinical practice, and it can be said that CAR is superior to the GPS/mGPS system for prognostic prediction.

CRP is a representative acute phase response protein whose levels rapidly increase in response to inflammation, and has been regarded as a definitive marker of systemic inflammation *in vivo* [45]. In clinical practice, it has been a most widely used index to evaluate the severity of the systemic inflammation or outcomes of various inflammation- related disorders. Approximate 90% of normal populations have a serum CRP concentration of less than 0.3 mg/dl, while cancer patients represent a significantly higher serum CRP level. CRP is mainly synthesized by liver and is strongly induced by pro-inflammatory cytokines, especially interleukin-6 (IL-6). Tumor tissue can trigger inflammatory response, further promotes the accumulation of inflammatory cells and the release of pro-inflammatory cytokines, resulting in an increased production of CRP by hepatocytes [46]. Simultaneously, some tumor cells, including ESCC and NSCLC, also have the ability to secret CRP alone and may contribute to the serum CRP level [47]. Therefore, cancers often occur along with increased CRP levels. Elevated CRP levels may contribute to create a favorable microenvironment for tumor cells proliferation and metastatization. Numerous epidemiological studies have showed that elevated CRP has been correlated with persistent fatigue, increased weight loss, low performance status and poor survival of cancer patients [48]. With the presence of elevated serum CRP concentration, serum level of vascular endothelial growth factor is also increased, which plays a critical role in promoting cancer angiogenesis and improving oxygen supply of cancer cells [49]. Moreover, CRP genetic polymorphism was associated with lymph node (LN) metastasis in several cancer types, including ESCC, NSCLC, and breast cancer [50]. The CRP 1846C > T polymorphism could be a novel predictor of LN involvement in ESCC, which was more accurate than computed tomography [51]. These findings indicated that CRP not only exists as an inflammatory marker, but also act as a prognostic predictor of malignant tumors.

ALB is also produced by the liver, which plays an important role in the maintenance of intravascular oncotic pressure, the transport of substances and the scavenging of free radicals. As one of the most common markers for assessing nutritional status, the serum ALB levels fall sharply during cancer progression because both malnutrition and systematic inflammation can suppress ALB synthesis [52]. During the malignant progress of tumors, tumor-related inflammatory cells are activated and secret several pro-inflammatory cytokines, including tumor necrosis factor, IL-1, IL-6, and IL-8. These cytokines can inhibit the ability of liver cells to generate ALB and increases the permeability of capillaries, which lead to a direct decrease of ALB concentration in the circulatory system [53]. Moreover, the development of micrometastases in liver tissue induces liver dysfunction and reduces the synthesis of ALB [54]. Thus, numerous studies have suggested that pretreatment serum ALB can be an effective prognostic indicator for cancer patients, as the low level of serum ALB predicts the worse prognosis.

Although both pretreatment CRP and ALB can be the independent prognostic factors of cancer patients, they are not perfect predictors because they can be easily influenced by some nontumor-related factors, such as diet, overhydration, and inflammation outside the tumor site. Several researches have revealed that the occurrence of decreased serum ALB levels, namely hypoalbuminemia, is secondary to the elevation of serum CRP, as many cancer patients with hypoalbuminemia already have high concentrations of serum CRP [55]. So CRP and ALB, represent two complementary statuses *in vivo*, are more appropriate to be combined as a composite parameter rather than being used as single predictors for prognosis of solid tumors.

Similar to other systematic review and meta-analysis, some limitations of this meta-analysis should be acknowledged. First, because the numbers of subgroup analyses dealing with each cancer type were less than 5, the evidence supporting the findings of the particular carcinomas might be less powerful. Moreover, due to the limited number of studies, we were unable to conduct subgroup analyses for certain types of cancer, such as OC, RCC, NSCLC and SCLC. Second, because no acknowledged threshold was available, different cut-offs to define “high” level of CAR were used in our included studies, which might also lead to clinical and statistical heterogeneity, and affect the availability of CAR as a predictive index in cancer prognosis to some degree. Third, the fact that researches with positive results tended to be published than works with null or negative outcomes, might induce reporting bias and potentially exaggerate the association between CAR and prognosis in solid tumors. Fourth, as all included papers were of East Asian origin, there may limitation on racial representation and the conclusions should be taken cautiously for other ethnic populations. Fifth, our meta-analysis primarily concentrated on pretreatment CAR, and the clinical significance of posttreatment CAR change, which may dynamic reflect the variation of the balance between host inflammatory response and nutritional status after therapy, is completed unclear. Finally, only evidence for correlation study was provided by the present meta-analysis, which could not be simply interpreted as causal relationship.

In conclusion, our meta-analysis suggested that elevated CAR is closely associated with poor survival including OS and DFS, as well some unfavorable clnilcopathological features in patients with solid tumors. Given its quickness, convenience, inexpensiveness, and reproducibility in clinical application, we believe that CAR can serve as a useful, effective index for the algorithm concerning the prognostic evaluation of cancer patients. However, due to the limitations of our meta-analysis, larger well-designed studies are recommended to validate the prognostic value of CAR in various tumors, and further explore the superiority of CAR over other traditional inflammatory markers by head to head comparisons.

## MATERIALS AND METHODS

### Literature search strategy

This meta-analysis was conducted according to the guidelines of the Preferred Reporting Items for Systematic Reviews and Meta-Analyses (PRISMA) [56]. A comprehensive electronic searching of PubMed, Embase, Web of Science, Cochrane Library databases, and China National Knowledge Infrastructure was conducted without language restrictions. We used combinations of the following key words: “C-reactive protein/albumin ratio or C reactive protein to albumin ratio or C-reactive albumin ratio or CRP/Alb ratio or CAR (all fields), cancer or tumor or malignancy or neoplasm or carcinoma (all fields), and prognosis or prognostic or survival or outcome (all fields)”. The final search was updated on Augest 2017. The citation lists of the included studies in this meta-analysis as well as any appropriate review articles were also screened for further relevant papers.

### Study selection criteria

Publications were eligible for this meta-analysis if they met all of the following criteria: (1) full papers with cohort design reported the relationship between pretreatment CAR and prognostic outcomes of solid tumors, such as OS, and DFS; (2) the patients with solid tumors were divided into two groups according to the CAR level, regardless of the cut-off value; (3) the HRs and 95% CIs for survival outcomes could be directly obtained from the original data or indirectly calculated from sufficient information. Studies were considered ineligible if they were reviews, conference abstracts, editorials, or letters, and they belonged to basic research and animal experiments. For multiple publications from the same authors or institutes with the same patient cohorts, only the most recent data were retained in the final analysis to avoid duplicate information.

### Data extraction and quality assessment

Two authors (JYW and WKT) independently reviewed the included studies and performed data extraction. Any disagreement between the reviewers was resolved by consensus. The following information was recorded for each study: first name of the authors, year of publication, study region, cancer type, primary treatment, duration period, follow-up time, sample number, cut-off selection, cut-off value, elevated CAR case number, clinical features, survival outcomes, HR estimation, and quality scores. The HRs and the corresponding 95% CIs were extracted from multivariate analyses where available, because it is more precise due to accounting for the confounding factors. Otherwise, HRs were extracted from the univariate analysis or calculated using the Kaplan–Meier survival curves [57].

The quality of each study was assessed by Newcattle-Ottawa Scale (NOS) according to the following categories: selection (four points), comparability (two points), and outcome of interest (three points) [58]. The total score of NOS ranged from 0 to 9, and we considered studies of six or more as high quality.

### Statistical analysis

All statistical analyses were performed by using STATA version 13.0 (STATA Corporation, College Station, TX, USA). Combined HRs and their 95% CIs were used to measure the impact of elevated CAR on survival. An observed HR greater than 1 indicated poor prognosis for patients with high CAR with its 95% CI exceeding 1. The statistical significance of the pooled HR was determined through *Z*–test, and *P* < 0.05 indicated statistical significance. Subgroup analyses were conducted according to cancer type (at least two trials must report the same outcome for the same cancer type; otherwise, they will be assigned to a subgroup designated “Others”), study region (“China”, “Japan” and “Korea”), primary treatment (“surgery”, “CT”, “CCRT” and “none reported [NR]”), clinical stage (“All stages”, “I–III”, and “advanced”), cut-off selection (“ROC”, “cutoff finder”, and “median”), and sample size (“< 200” and “≥ 200”). Moreover, a subgroup analysis based on cutoff values were also performed, namely “< 0.1” and “≥ 0.1”, because 0.1 was the median of the cut-off values to define “high” level of CAR among the included studies. Meta-regression analysis was also performed to determine the potential sources of heterogeneity. The ORs and the corresponding 95% CIs were used to estimate the correlation between CAR and clinicopathological features. All statistical tests were two sided.

Statistical heterogeneity was examined by the Cochran’s Q statistic qualitatively, and the I^2^ metric quantitatively (I^2^ ≤ 25%, no heterogeneity; 25% < I^2^ < 50%, moderate heterogeneity; and I^2^ ≥ 50%, extreme heterogeneity) [59]. When significant heterogeneity had been observed among the studies (*P* < 0.10 or I^2^ > 50%), the pooled HR estimation of each study was calculated using a random-effects model (DerSimonian and Laird method). Otherwise, a fixed-effects model was applied (Mantel–Haenszel method) [60]. Sensitivity analysis was conducted by sequentially omitting each individual study to validate the stability of the meta-analysis outcomes. The effect of potential publication bias on the outcomes was quantitatively evaluated through Begg’s and Egger’s asymmetry tests [61], and was visually evaluated using funnel plots. If significant publication bias existed, trim and fill method was performed to validate the robust of the meta-analysis results [62]. The statistical significance of Begg’s and Egger’s tests was defined as *P* < 0.10.

## SUPPLEMENTARY MATERIALS TABLE




