# Supplementary material for: Clinicopathologic and prognostic significance of C-reactive protein/albumin ratio in patients with solid tumors: an updated systemic review and meta-analysis
Source: Oncotarget. 2018 Jan 11;9(17):13934–47. doi: 10.18632/oncotarget.24172 (PMC5862627; doi:10.18632/oncotarget.24172)
Supplement: Supplementary file 1 [file oncotarget-09-13934-s001.pdf]

Clinicopathologic and prognostic significance of C-reactive protein/albumin ratio in patients with solid tumors: an updated systemic review and meta-analysis

SUPPLEMENTARY MATERIALS

Supplementary Table 1: Main characteristic of the eligible studies. See Supplementary_Table_1
