# Supplementary material for: Clinicopathologic and prognostic significance of C-reactive protein/albumin ratio in patients with solid tumors: an updated systemic review and meta-analysis
Source: Oncotarget. 2018 Jan 11;9(17):13934–47. doi: 10.18632/oncotarget.24172 (PMC5862627; doi:10.18632/oncotarget.24172)
Supplement: Supplementary file 2 [file oncotarget-09-13934-s002.docx]

**Table 1** **Main characteristic of the eligible studies**

| **Study** | **Region** | **Duration** | **Cancer type** | **Clinical stage** | **Primary treatment** | **Follow up**  **(month)** | **Number** | **Cut off selection** | **Cut off** | **Elevated**  **CAR (%)** | **Analysis** | **Outcome** | **Language** | **Quality** |
| --- | --- | --- | --- | --- | --- | --- | --- | --- | --- | --- | --- | --- | --- | --- |
| Sun P (2017) [21] | China | 2008-2011 | NPC | Ⅳ | CT | median 15 (1-66) | 148 | Cutoff finder | 0.189 | 70 (47.3) | Multivariate | OS | English | 8 |
| Yu ST (2017) [22] | China | 2006-2011 | LSCC | Ⅰ-Ⅳ | Surgery | median 77 (66-94) | 129 | ROC | 0.047 | 73 (56.6) | Multivariate | OS, DFS | English | 7 |
| Liu YB (2017) [23] | China | 2006-2012 | OC | Ⅰ-Ⅳ | Surgery | Until 2014.12 | 200 | ROC | 0.68 | 69 (34.5) | Multivariate | OS | English | 8 |
| Guo SJ (2017) [24] | China | 2000-2012 | RCC | Ⅰ-Ⅳ | Surgery | Until 2015.11 | 570 | ROC | 0.08 | 393 (68.9) | Multivariate | OS, DFS | English | 8 |
| Koh YW (2017) [25] | Korea | 2002-2011 | NSCLC | Ⅰ-Ⅳ | CT | median 9 (1-74) | 165 | Cutoff finder | 0.195 | 108 (65.5) | Multivariate | OS | English | 9 |
| Haruki K (2016) [26] | Japan | 2001-2011 | PC | Ⅰ-Ⅳ | Surgery | NR | 113 | ROC | 0.03 | 58 (51.3) | Multivariate | OS, DFS | English | 8 |
| Wu MW (2016) [27] | China | 2011-2014 | PC | Ⅲ-Ⅳ | CT | until 2015.9 | 233 | Cutoff finder | 0.54 | 74 (31.8) | Multivariate | OS | English | 8 |
| Liu ZQ (2016) [28] | China | 2010-2015 | PC | Ⅰ-Ⅳ | NR | median 9 | 386 | ROC | 0.18 | 91 (23.6) | Multivariate | OS | English | 8 |
| Lee JM (2016) [29] | Korea | 2011-2014 | PC | Ⅰ-Ⅳ | CT | until 2015.8 | 82 | ROC | 0.5 | 40 (48.8) | Multivariate | OS | English | 7 |
| Shibutani M (2016) [30] | Japan | 2005-2010 | CRC | Ⅰ-Ⅳ | CT | median 21 (3-73) | 99 | ROC | 0.183 | 36 (36.4) | Multivariate | OS | English | 7 |
| Ishizuka M (2016) [31] | Japan | 2006-2013 | CRC | Ⅰ-Ⅳ | Surgery | median 30 | 627 | ROC | 0.038 | 366 (58.4) | Multivariate | OS | English | 8 |
| Tominaga T (2016) [32] | Japan | 2005-2014 | CRC | Ⅲ | CT | NR | 136 | ROC | 0.1 | 30 (22.1) | Multivariate | DFS | English | 8 |
| Ni XF (2016) [33] | China | 2010-2015 | CRC | Ⅳ | CT | median 12 (0.4-67) | 148 | ROC | 0.6712 | 45 (30.4) | Multivariate | OS | English | 7 |
| Zhang Y (2016) [34] | China | 2009-2012 | NPC | Ⅰ-Ⅳ | CCRT | median 50 (1-76) | 1572 | ROC | 0.05 | 614 (39.1) | Multivariate | OS, DFS | English | 7 |
| He SS (2016) [35] | China | 2000-2013 | NPC | Ⅰ-Ⅳ | CCRT | median 46 (1-188) | 2685 | ROC | 0.064 | 1022 (38.1) | Multivariate | OS | English | 9 |
| Li JP (2016) [36] | China | 2009-2010 | NPC | Ⅰ-Ⅳ | CCRT | until 2015.1 | 409 | Median | 0.003 | 228 (55.7) | Multivariate | OS | English | 8 |
| Toiyama Y (2016) [37] | Japan | 2001-2011 | GC | Ⅰ-Ⅲ | Surgery | median 48 (40-54) | 384 | ROC | 0.051 | 163 (42.4) | Multivariate | OS, DFS | English | 7 |
| Park HC (2016) [38] | Korea | 2004-2011 | OSCC | Ⅰ-Ⅳ | Surgery | median 36 | 40 | ROC | 0.085 | 13 (32.5) | Multivariate | OS | English | 8 |
| Li M (2016) [39] | China | 2011-2014 | HCC | Ⅰ-Ⅳ | Surgery | median 36 (19-55) | 178 | ROC | 0.46 | 97 (54.5) | Multivariate | OS | Chinese | 7 |
| Xu XL (2015) [40] | China | 2000-2010 | ESCC | Ⅰ-Ⅲ | Surgery | median 50 (11-88) | 468 | ROC | 0.5 | 87 (18.6) | Multivariate | OS | English | 8 |
| Wei XL (2015) [41] | China | 2006-2010 | ESCC | Ⅰ-Ⅳ | Surgery | median 36 (1-96) | 423 | ROC | 0.095 | 147 (34.8) | Multivariate | OS | English | 8 |
| Liu XC (2015) [42] | China | 2005-2010 | GC | Ⅰ-Ⅲ | Surgery | median 25 (1-76) | 455 | ROC | 0.25 | 302 (66.4) | Multivariate | OS | English | 8 |
| Kinoshita A (2015) [43] | Japan | 2005-2012 | HCC | Ⅰ-Ⅳ | Surgery | median 18 (1-88) | 186 | ROC | 0.037 | 102 (54.8) | Multivariate | OS | English | 9 |
| Zhou T (2015) [44] | China | 2006-2011 | SCLC | Limited-  extensive | CT | median 29 (1-116) | 367 | Cutoff finder | 0.441 | 128 (34.8) | Multivariate | OS | English | 8 |

CAR C-reactive protein/albumin ratio; NR none reported; LSCC laryngeal squamous cell carcinoma; OC ovarian cancer; RCC renal cell carcinoma; NSCLC non small cell lung cancer; PC pancreatic cancer; CRC colorectal cancer; NPC nasopharyngeal carcinoma; GC gastric cancer; OSCC oral squamous cell carcinoma; HCC hepatocellular carcinoma; ESCC esophageal squamous cell carcinoma; SCLC small cell lung cancer; OS overall survival; DFS disease-free survival; CT chemotherapy; CCRT concurrent chemoradiotherapy; ROC the receiver operating characteristic.
